# Association of COVID-19 with Intracranial Hemorrhage during Extracorporeal Membrane Oxygenation for Acute Respiratory Distress Syndrome: A 10-Year Retrospective Observational Study

**DOI:** 10.3390/jcm11010028

**Published:** 2021-12-22

**Authors:** Tobias Pantel, Kevin Roedl, Dominik Jarczak, Yuanyuan Yu, Daniel Peter Frings, Barbara Sensen, Hans Pinnschmidt, Alexander Bernhardt, Bastian Cheng, Iris Lettow, Manfred Westphal, Patrick Czorlich, Stefan Kluge, Marlene Fischer

**Affiliations:** 1 Department of Neurosurgery, University Medical Center Hamburg-Eppendorf, 20246 Hamburg, Germany; tobiasfabian.pantel@uke.de (T.P.); westphal@uke.de (M.W.); p.czorlich@uke.de (P.C.); 2 Department of Intensive Care Medicine, University Medical Center Hamburg-Eppendorf, 20246 Hamburg, Germany; k.roedl@uke.de (K.R.); d.jarczak@uke.de (D.J.); d.frings@uke.de (D.P.F.); b.sensen@uke.de (B.S.); s.kluge@uke.de (S.K.); 3 Department of Anesthesiology, University Medical Center Hamburg-Eppendorf, 20246 Hamburg, Germany; yuanyuan.yu@stud.uke.uni-hamburg.de; 4 Institute of Medical Biometry and Epidemiology, University Medical Center Hamburg-Eppendorf, 20246 Hamburg, Germany; h.pinnschmidt@uke.de; 5 Department for Cardiovascular Surgery, University Medical Center Hamburg-Eppendorf, 20246 Hamburg, Germany; al.bernhardt@uke.de; 6 Department of Neurology, University Medical Center Hamburg-Eppendorf, 20246 Hamburg, Germany; b.cheng@uke.de (B.C.); i.lettow@uke.de (I.L.)

**Keywords:** COVID-19, extracorporeal membrane oxygenation, intracerebral hemorrhage, hemorrhagic stroke, neurologic complications, acute respiratory distress syndrome

## Abstract

Extracorporeal membrane oxygenation (ECMO) is potentially lifesaving for patients with acute respiratory distress syndrome (ARDS) but may be accompanied by serious adverse events, including intracranial hemorrhage (ICRH). We hypothesized that ICRH occurs more frequently in patients with COVID-19 than in patients with ARDS of other etiologies. We performed a single-center retrospective analysis of adult patients treated with venovenous (vv-) ECMO for ARDS between January 2011 and April 2021. Patients were included if they had received a cranial computed tomography (cCT) scan during vv-ECMO support or within 72 h after ECMO removal. Cox regression analysis was used to identify factors associated with ICRH. During the study period, we identified 204 patients with vv-ECMO for ARDS, for whom a cCT scan was available. We observed ICRH in 35.4% (*n* = 17/48) of patients with COVID-19 and in 16.7% (*n* = 26/156) of patients with ARDS attributable to factors other than COVID-19. COVID-19 (HR: 2.945; 95%; CI: 1.079–8.038; *p* = 0.035) and carboxyhemoglobin (HR: 0.330; 95%; CI: 0.135–0.806; *p* = 0.015) were associated with ICRH during vv-ECMO. In patients receiving vv-ECMO, the incidence of ICRH is doubled in patients with COVID-19 compared to patients suffering from ARDS attributable to other causes. More studies on the association between COVID-19 and ICRH during vv-ECMO are urgently needed to identify risk patterns and targets for potential therapeutic interventions.

## 1. Introduction

The number of patients presenting with acute respiratory distress syndrome (ARDS) has increased substantially since the novel severe acute respiratory syndrome coronavirus type 2 (SARS-CoV-2) has spread worldwide and caused the ongoing COVID-19 pandemic [1]. At the same time, the demand for adjunctive treatment options and advanced supportive care has renewed interest in extracorporeal membrane oxygenation (ECMO) and rapidly increased its use [2].

Current guidelines recommend venovenous ECMO (vv-ECMO) in ARDS patients with hypoxemia or impaired decarboxylation refractory to supportive care, including but not limited to mechanical ventilation, prone positioning, and restrictive fluid management [3]. Similarly, ECMO should be considered in COVID-19-associated ARDS with hypoxemia or hypercapnia not responding to adjunctive treatment [2,4]. Although providing lifesaving pulmonary support, ECMO-related complications may be associated with fatal outcomes [5,6,7]. Studies of intracranial adverse events associated with ECMO therapy from the pre-COVID-19 era are manifold. Intracranial hemorrhage (ICRH) represents the most devastating complication, with a reported incidence of 3.1% to 12.3%, leading to a mortality rate as high as 73% [5,8,9,10].

To date, data are limited regarding intracranial complications and neuroimaging characteristics in patients requiring vv-ECMO for ARDS and including COVID-19-associated ARDS. Importantly, most studies on ICRH during ECMO for ARDS were carried out in the pre-COVID-19 era [5,7,9,11]. We aimed to analyze the incidence of ICRH during vv-ECMO in ARDS patients with and without COVID-19 and to characterize bleeding patterns on neuroimaging. We hypothesized that ICRH occurs more frequently in ARDS patients with COVID-19 compared with patients with ARDS from causes other than SARS-CoV-2 infection. In addition, we sought to identify factors associated with ICRH to improve individual risk stratification during ECMO and ICU treatment.

## 2. Materials and Methods

### 2.1. Ethics Statement

Ethical approval for this study was obtained from the ethics committee of the Hamburg Chamber of Physicians (No.: WF-046/21). The need for informed consent was waived because of the retrospective nature of the study and the use of routine clinical data. The study was performed in accordance with the Declaration of Helsinki and its later amendments.

### 2.2. Setting, Design, and Participants

This retrospective observational cohort study was performed at the Department of Intensive Care Medicine, University Medical Center Hamburg-Eppendorf, Hamburg, Germany. The Department of Intensive Care Medicine is part of a tertiary care hospital with the majority of patients referred to from other health care institutions for evaluation of ECMO indication. Patients who received high-flow vv-ECMO for acute respiratory failure attributable to ARDS between January 2011 and April 2021 were identified from electronic health records (Integrated Care Manager, Version 10.01, Dräger AG, Lübeck, Germany and Soarian^®^ Clinicals, Siemens Healthcare GmbH, Erlangen, Germany). We included patients for whom brain imaging by computed tomography (CT) was performed during vv-ECMO support or within the first 72 h after ECMO removal. Additional inclusion criteria were ages of at least 18 years and vv-ECMO support of at least 6 h. Patients were excluded from the final analysis if they had a documented acute brain injury, including any type of ICRH, ischemic stroke, or hypoxic-ischemic brain injury before the initiation of vv-ECMO.

### 2.3. Neurological Evaluation and Brain Imaging

During vv-ECMO support, patients were routinely monitored for clinical signs of neurological deterioration such as newly occurring abnormal pupillary status or other neurological signs accessible to clinical examination in sedated patients. Furthermore, failure to regain consciousness or other neurological deficits apparent were re-assessed after discontinuation of sedation. Abnormal clinical neurological findings were additionally assessed by a board-certified neurologist. In case of persisting neurological abnormalities, a cranial CT scan was initiated for further diagnostics.

We retrospectively reviewed cranial CT scans of all patients included in the study. All CT data were reviewed independently by one neurosurgeon (TP) and one neurointensivist (MF), who were blinded for the patients’ diagnoses including the presence of acute brain injury. When these two did not concur, cranial CT scans were reviewed by another experienced neurosurgeon (PC). Cranial CT scans were visually assessed for the presence of intraparenchymal hemorrhage, intraventricular hemorrhage (IVH), subarachnoid hemorrhage, and subdural or epidural hematoma. For the purpose of statistical analyses, all types of hemorrhage were summarized as ICRH. Computed tomography scan evaluation was performed with Centricity Universal Viewer Zero Footprint Client^®^ (GE Healthcare, Boston, MA, USA).

Volumes of intraparenchymal hemorrhage and IVH were assessed with Origin Server 3.1 (Brainlab, Munich, Germany) using the iPlan function and presented in milliliters. Volumetric analysis was performed based on the first cranial CT scan, where the onset of intraparenchymal hemorrhage or IVH was documented. In case of multiple bleeding spots, single volumes were summed up. The location, distribution, and extent of intraparenchymal hemorrhage were categorized according to the classification proposed by Prinz et al., and ECMO-associated bleeding events were categorized as lobar intraparenchymal hemorrhage with, I/A, or without IVH, II/B, and multiple small bleeding spots, III/C [6]. The classification has been linked with clinical outcome with type I/A hemorrhage resulting in poorest outcomes.

### 2.4. ECMO and Coagulation Management

Patients with severe hypoxemic and/or hypercapnic respiratory failure in combination with severe respiratory acidosis refractory to adjunctive therapies received vv-ECMO (CARDIOHELP-System Maquet GmbH, Rastatt, Germany; Novalung, Fresenius Medical Care, Bad Homburg, Germany; Stöckert Centrifugal Pump Console, LivaNova Deutschland GmbH, Munich, Germany). Criteria for the initiation of vv-ECMO support were based on the guidelines of the Extracorporeal Life Support Organization and national recommendations [3,12]. Details of the management of ARDS and vv-ECMO therapy are provided in Additional Text S1.

### 2.5. Statistical Analysis

Data retrieval and collection are elaborated in Additional Text S1. Data are presented as median with interquartile range (continuous variables) or absolute and relative numbers (categorical variables). Demographic and clinical variables were compared between patients with and without ICRH with Mann–Whitney-U tests, chi-square tests, or Fisher’s exact tests, as appropriate. Continuous variables were graphically checked for normal distribution using histograms.

For further multivariable Cox regression analyses, we identified the candidate explanatory variables COVID-19, sex, Charlson Comorbidity Index, highest carboxyhemoglobin (COHb) during vv-ECMO or before ICRH, delta PaCO_2_ (difference in arterial partial pressure of carbon dioxide before vv-ECMO placement and PaCO_2_ 24 h after start of vv-ECMO), lowest platelets during vv-ECMO or before ICRH, lowest fibrinogen during vv-ECMO of before ICRH, highest activated partial thromboplastin time during vv-ECMO or before ICRH, maximum cannula size, Sequential Organ Failure Assessment (SOFA) score on admission, and year of vv-ECMO implantation. The selected variables were considered clinically relevant or had been linked with ICRH in previous studies [5,9,10,13]. Relationships among these potential explanatory variables, the endpoint ICRH, and the associated event time variables (days until ICRH) were examined by nonlinear categorical principal components analysis (CATPCA), evaluating the relative importance of the variables within their multivariable setting and the strength of their associations based on their loadings and vector angles in the first two dimensions extracted by CATPCA [14]. The variance inflation factors of the explanatory variable candidates were also computed and found to be less than 3 for any variable. The variables Charlson Comorbidity Index, highest COHb, delta PaCO_2_, lowest platelets, and highest activated partial thromboplastin time were transformed to their natural logarithm before further analyses because they displayed right-skewed data distributions. All explanatory variables as described above were entered in multivariable Cox regression analyses. The effects of the independent variable of primary interest “COVID-19” were adjusted for confounding with the variable “year of ECMO implantation” by always forcing both variables into the cox regression models. The remaining variables were selected stepwise-backward with “ICRH during vv-ECMO” as the dependent variable and the time between vv-ECMO start and diagnosis of ICRH as response variable. Cox regression analyses were performed separately on data from 2020 to 2021. We present hazard ratios with 95% confidence intervals and *p* values resulting from these analyses. Statistical analyses were performed with IBM SPSS Statistics (Version 27, IBM Corporation, Armonk, New York, NY, USA). Figures were designed with Prism 9 for mac OS (GraphPad Software Inc., San Diego, CA, USA).

## 3. Results

### 3.1. Study Population

**Figure 1:**
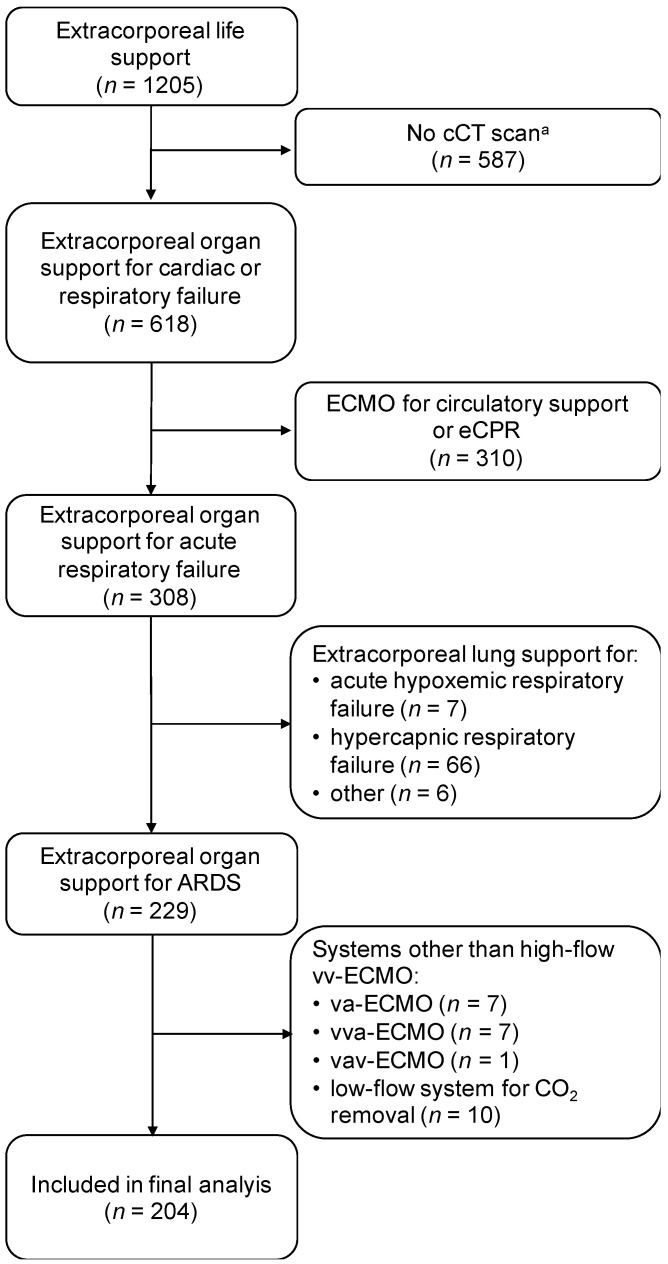
Flow diagram of patient selection during the study period (2011–2021). ARDS: acute respiratory distress syndrome. CCT: cranial computed tomography. CO_2_: carbon dioxide. Vv-/va-/vva-/vav-ECMO: veno-venous/veno-arterial/veno-veno-arterial/veno-arterio-venous extracorporeal membrane oxygenation. eCPR: extracorporeal cardiopulmonary resuscitation. ^a^ Patients, who did not receive a cCT scan during ECMO support or within the first 72 h after ECMO removal, were excluded.

Between January 2011 and April 2021, a total of 1205 patients received extracorporeal organ support for cardiac or respiratory failure at our department. Cerebral CT scans during or immediately after ECMO treatment were available from 618 patients (51.3%). Of these, 204 patients (16.9%) were treated with high-flow vv-ECMO systems for ARDS and were included for further analyses (Figure 1). Throughout the study period, high-flow vv-ECMO systems were used in a total number of 402 patients with acute respiratory failure. One hundred and fifty-six patients suffered from non-COVID-19-associated ARDS and received vv-ECMO support from January 2011 to April 2021 (Table 1). Another 48 patients were diagnosed with COVID-19-associated ARDS and had vv-ECMO therapy from March 2020 to April 2021. The baseline demographic and clinical characteristics of the study population are presented in Table 1.

### 3.2. Intracranial Hemorrhage

**Figure 2:**
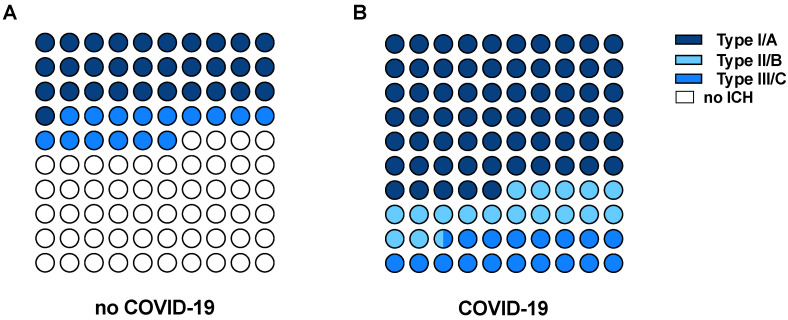
Types of intraparenchymal hemorrhage according to the classification proposed by Prinz and colleagues [6]. Data are given in % for patients without (**A**) and with COVID-19-associated ARDS (**B**). No intraparenchymal hemorrhage (ICH) includes patients with intraventricular or subarachnoid hemorrhage, epidural or subdural hematoma.

Patients with and without ECMO-related ICRH were comparable with regard to baseline demographic and clinical characteristics, as displayed in Table 1. Details of blood gas analyses before and after vv-ECMO support are given in Table S1. We found acute ICRH in 43 patients (21.1%) who had a cranial CT scan during vv-ECMO or shortly after ECMO removal. All ICRH occurred during the ICU stay. The median time between ICU admission and ICRH was 10 days (3–18), and the median time between ECMO initiation and ICRH was 7 days (3–12). Regarding the total number of patients who received vv-ECMO for acute respiratory failure throughout the study period, the incidence of ICRH is 10.7% (*n* = 43/402). Table S2 shows vital signs, settings of ECMO and mechanical ventilation, blood gas analyses, and coagulation parameters at the time of diagnosis of ICRH. Intraparenchymal hemorrhage was the predominant type of ICRH, with an incidence of 67.4% (*n* = 29/43; Figure 2). IVH associated with intraparenchymal hemorrhage was diagnosed in 18 of 43 cases (41.9%). Epidural, subdural, or sulcal subarachnoid hematoma was present in 37.2% (*n* = 16/43) of all patients with ICRH. Type I/A hemorrhage was found in 44.2% (*n* = 19/43), representing the predominant bleeding type. All patients with type I/A hemorrhage died during the ICU stay (Figure S1). The median volume of intraparenchymal hemorrhage was 69 (53–120) ml and 39 (30–51) ml for IVH. A detailed overview of all bleeding characteristics is given in Table 2. Surgical treatment was performed as microsurgical hematoma evacuation in only one case. Patients with ICRH had an overall intensive care unit mortality of 83.7%.

### 3.3. Factors Associated with ICRH during vv-ECMO

A diagnosis of COVID-19 (HR: 2.945; 95% CI: 1.079–8.038; *p* = 0.035) and lower levels of COHb (HR: 0.330; 95% CI: 0.135–0.806; *p* = 0.015) were associated with ICRH during vv-ECMO support. For a full list of the variables included in the initial model and details on the Cox regression analysis see Table 3. The probability of experiencing an ICRH for patients with and without COVID-19 is presented in Figure S2.

We performed an additional analysis for patients who received vv-ECMO in the years 2020 and 2021 (*n* = 56). Carboxyhemoglobin (HR: 0.016; 95% CI: 0.002–0.123; *p* < 0.001), fibrinogen (HR: 7.708; 95% CI: 2.238–26.549; *p* = 0.001), and SOFA score (HR: 0.840; 95% CI: 0.735–0.959; *p* = 0.010) were associated with ICRH in this subgroup of patients (Table S3).

### 3.4. ICRH in Patients with COVID-19

During the study period, a total of 89 patients received vv-ECMO for COVID-19-associated ARDS. A CT scan was available from 48 participants showing ICRH in 17 patients (35.4%). Demographic and clinical characteristics for patients with ICRH are presented in Table S4. All patients showed intraparenchymal hemorrhage: 100% (*n* = 17/17). Eight patients (47.1%) had an IVH, and another eight (47.1%) had epidural or subdural hematoma or sulcal subarachnoid hemorrhage during vv-ECMO therapy. We found type I/A intraparenchymal hemorrhage in a majority of patients (*n* = 11/17, 64.7%) with COVID-19-associated ARDS (Figure 2). Examples of different subtypes of intraparenchymal hemorrhage are presented in Figure S3. The median volume of intraparenchymal hemorrhage was 45 (20–81) mL and 45 (21–92) mL for IVH. No neurosurgical intervention was performed in any of these cases. A detailed overview of all bleeding patterns is shown in Table 2.

## 4. Discussion

To the best of our knowledge, this is the first study to present intracranial bleeding events in patients with and without COVID-19-associated ARDS who receive vv-ECMO. The main findings of our study are as follows: (1) Over 10 years, we found ICRH in 21% of ARDS patients who had a brain CT scan during vv-ECMO support. (2) The majority of intracranial bleeding events were categorized as grade I/A in our study population [6]. (3) ICRH occurred in 35.4% of patients with and 16.7% of patients without COVID-19-associated ARDS during vv-ECMO support. (4) COVID-19 and low COHb levels were associated with ICH in multivariable Cox regression analysis.

ECMO has evolved as a promising technique in the management of ARDS refractory to mechanical ventilation and adjunctive treatment [3,15]. Intracranial bleeding events are among the most feared ECMO-related complications and are associated with unacceptably high mortality [5,6,10]. The reasons for poor outcomes in patients with intracranial complications are multifactorial. First, time to diagnosis is frequently prolonged by impeded neurological assessment in regularly sedated patients. Second, once diagnosed, therapeutic options for intracranial hemorrhagic events are limited. One crucial factor that conflicts with neurosurgical interventions is the anticoagulation required during ECMO [3]. This aspect seems even more relevant in patients with COVID-19, as recent studies indicate a significant increase in life-threatening thromboembolic complications as a reaction to endothelial activation, proinflammatory processes, and interaction with hemostatic factors [16,17,18].

Studies of intracranial bleeding during vv-ECMO reported incidences up to 12.3%, with intraparenchymal hemorrhage as the predominant subtype [6,7,8,10]. We observed ICRH in 10.7% of all patients who received high-flow systems for acute respiratory failure throughout the study period; a number that is comparable with previous reports. For this study, we included only patients who had a brain CT scan during or early after ECMO treatment. The strict preselection of patients might explain the higher number of ICRH in our study population. Interestingly, we found higher rates of ICRH in patients with COVID-19-associated ARDS, as compared with ARDS from causes other than SARS-CoV-2 infection. This effect remained significant in multivariable analysis after adjustment for factors linked with ICRH during ECMO previously [5,9,11,13,19]. In a multicenter registry analysis of critically ill patients with COVID-19, 56% of patients with hemorrhagic stroke were on ECMO support, a finding that affirms the association between ICRH and ECMO for COVID-19 [20].

In a retrospective propensity-matched cohort study, Lang et al. observed intraparenchymal hemorrhage in 19% of patients with COVID-19-associated ARDS compared with 11% without COVID-19 [19]. This difference was not statistically significant, but notably, Lang et al.’s study included ARDS patients with and without ECMO, limiting the comparability with our results [19]. Yet, with 19.1%, we found a similar rate of ICRH relating to the total number of patients with vv-ECMO for COVID-19-associated ARDS during the study period.

COVID-19 is characterized by a hypercoagulable state resulting in an increased risk of thromboembolic events [18,21]. Consequently, more aggressive anticoagulation strategies have been proposed for patients with COVID-19 [18,22]. Because of COVID-19-associated hypercoagulopathy, there is an ongoing debate on anticoagulation targets for patients with COVID-19 requiring ECMO [16,18,23]. Whether more-intense anticoagulation might benefit this patient population remains a matter of controversy [23]. For patients with COVID-19, we did not use anticoagulation targets different from patients without COVID-19-associated ARDS. Therefore, a link between more aggressive anticoagulation and ICRH seems implausible. COVID-19 relates to endothelial dysfunction resulting in a prothrombotic state in most patients [18]. A differential interplay between COVID-19-associated coagulopathy and ECMO-related shear stress might increase susceptibility to hemorrhagic complications.

Recently, Prinz et al. proposed a classification for imaging features of intraparenchymal hemorrhage in patients with ECMO [6]. This is the first attempt to categorize patients with similar patterns of intraparenchymal hemorrhage and to allow for an outcome stratification based on neuroimaging. Type I/A intraparenchymal hemorrhage includes features of greater lobar hemorrhage with consecutive intraventricular bleeding, frequently accompanied by mass effects. Thus, a type I/A hemorrhage comprises different aspects of acute brain injury associated with high mortality. Although the result lacks statistical significance, we observed more type I/A intraparenchymal hemorrhages in the COVID-19 subgroup compared with non-COVID-19 patients. Both the increased incidence of ICRH and the distribution of bleeding patterns indicate heightened risk of ECMO-associated intraparenchymal hemorrhage with mass effects in patients with COVID-19.

For multivariable analysis of factors associated with ICRH, we chose parameters reported in the context of acute brain injury during vv-ECMO in recent studies [5,9,11,13,19]. As opposed to previous reports, ours did not find a statistical association between coagulopathy or changes of carbon dioxide and ICRH. We used COHb as a surrogate for hemolysis [24,25]. Surprisingly, we found lower levels of COHb to be associated with ICRH. There are contradictory findings on COHb in critically ill patients. Elevated COHb was associated with higher overall in-hospital mortality in a prospective observational study [26]. In line with our results very low values of COHb have been observed in non-survivors in a medical intensive care unit [27].

### Limitations and Strengths

Our study has several limitations that must be addressed. First, we present findings from a single-center retrospective study limiting the generalizability of our results. The nature of our study design does not allow conclusions about future management of vv-ECMO for ARDS in patients with and without COVID-19. However, our findings may contribute to the understanding of risk patterns for ICRH during ECMO support. Second, we included only patients who received a cranial CT scan during vv-ECMO treatment or within the 72 h immediately following ECMO removal. Therefore, we may have overestimated the incidence of ICRH. When reviewing the total number of patients who received vv-ECMO for acute respiratory failure throughout the study period, we found an incidence of ICRH of 10.7%. Importantly, the strict exclusion of patients without neuroimaging is also a strength of our study, as it reduces the probability of bias attributable to undetected brain injury. Still, we may have missed patients with ICRH before ECMO initiation, since not all patients at our center receive a cranial CT scan prior to ECMO start. Third, the change in ARDS management over time may have biased our results [28]. We included patients with ARDS from causes other than COVID-19 from 2011, whereas the first case of COVID-19 received ECMO for acute respiratory failure at our center in April 2020. However, we adjusted for the confounding effect of the year of ECMO support by forcing the variable into the regression equation.

One strength of this study is its inclusion of only patients treated for ARDS with a high-flow vv-ECMO system. By excluding patients who received ECMO for circulatory support or for respiratory failure caused by factors other than ARDS, we selected a homogenous study population allowing for acceptable comparability of incidences and factors associated with ICRH between patients with and without COVID-19.

## 5. Conclusions

Despite similar anticoagulation targets, we found ICRH in twice as many patients with COVID-19 than in patients suffering from ARDS attributable to other causes. ICRH during ECMO support is a devastating condition frequently resulting in death. Therefore, more studies on the association between COVID-19 and ICRH during vv-ECMO are urgently needed to identify risk patterns and targets for potential therapeutic interventions.

## Figures and Tables

**Table 1:** Demographic and clinical characteristics.

	No Intracranial Hemorrhage	Intracranial Hemorrhage	*p*
	(*n* = 161)	(*n* = 43)	
**Age, years**	55 (47–64)	56 (47–63)	0.587
**Gender (female)**	55 (34.2)	15 (34.9)	0.929
**Body Mass Index, kg/m^2^**	27.8 (24.5–32.1)	27.7 (23.4–32.4)	0.604
**Myocardial infarction**	19 (11.8)	4 (9.3)	0.790
**Chronic heart failure**	2 (1.2)	3 (7.0)	0.064
**Peripheral vascular disease**	6 (3.7)	1 (2.3)	1.000
**Cerebrovascular disease**	5 (3.1)	2 (4.7)	0.64
**Dementia**	1 (0.6)	0 (0.0)	1.000
**COPD**	24 (14.9)	1 (2.3)	0.033
**Connective tissue disease**	15 (9.3)	3 (7.0)	0.770
**Peptic ulcer disease**	8 (5.0)	1 (2.3)	0.688
**Chronic kidney disease**	7 (4.3)	2 (4.7)	1.000
**Leukemia**	2 (1.2)	1 (2.3)	0.510
**Lymphoma**	7 (4.3)	6 (14.0)	0.022
**Liver disease**			0.088
Mild	10 (6.2)	0 (0.0)	
Moderate to severe	3 (1.9)	0 (0.0)	
**Diabetes mellitus**			0.208
Uncomplicated	32 (19.9)	5 (11.6)	
End-organ damage	1 (0.6)	0 (0.0)	
**Solid tumor**			0.328
Localized	18 (11.2)	4 (9.3)	
Metastatic	4 (2.5)	0 (0.0)	
**AIDS**	3 (1.9)	1 (2.3)	1.000
**Charlson Comorbidity Index**	2 (1–4)	2 (1–3)	0.769
**COVID-19**	31 (19.3)	17 (39.5)	0.005
**SAPS II on admission**	42 (34–50)	41 (36–52)	0.733
**SOFA on admission**	12 (11–15)	12 (8–13)	0.056
**Duration of mechanical ventilation, days**	24 (13–36)	14 (8–30)	0.010
**Inhalational nitric oxide**	98 (59.6)	25 (58.1)	0.745
**RRT**	117 (72.7)	29 (67.4)	0.499
**RRT duration, days**	10 (1–24)	9 (0–16)	0.349
**Primary indication for ECMO**			0.125
Oxygenation	151 (93.8)	43 (100)	
Decarboxylation	10 (6.2)	0 (0.0)	
**Device**			0.939
Venovenous ECMO	143 (83.2)	36 (83.7)	
iLA activve^®^ (pump-assisted)	27 (16.8)	7 (16.3)	
**Year of ECMO implantation**			0.038
**2011**	3 (1.9)	0 (0.0)	
**2012**	4 (2.5)	1 (2.3)	
**2013**	12 (7.5)	4 (9.3)	
**2014**	8 (5.0)	2 (4.7)	
**2015**	11 (6.8)	2 (4.7)	
**2016**	26 (16.1)	2 (4.7)	
**2017**	13 (8.1)	1 (2.3)	
**2018**	26 (16.1)	9 (20.9)	
**2019**	17 (10.6)	2 (4.7)	
**2020**	36 (22.4)	12 (27.9)	
**2021 (before April 30, 2021)**	5 (3.1)	8 (18.6)	
**Maximum cannula size, Fr**	24 (23–25)	24 (23–25)	0.889
**Minimum cannula size, Fr**	17 (17–17)	17 (17–19)	0.255
**ASS before ECMO**	23 (14.3)	7 (16.3)	0.743
**Other platelet inhibitor before ECMO**			0.867
None	158 (98.8)	42 (97.7)	
Clopidogrel	1 (0.6)	0 (0.0)	
Prasugrel	0 (0.0)	1 (2.3)	
Ticagrelor	1 (0.6)	0 (0.0)	
**Anticoagulation or coagulopathy before ECMO**			0.562
No	127 (78.9)	33 (76.7)	
Yes	27 (16.8)	9 (20.9)	
Unknown	7 (4.3)	1 (2.3)	
**Heart rate ^a^, bpm**	105 (92–119)	108 (94–125)	0.521
**Systolic blood pressure ^a^, mmHg**	120 (105–134)	123 (105–142)	0.372
**Diastolic blood pressure ^a^, mmHg**	54 (47–65)	55 (44–64)	0.619
**Mean arterial pressure ^a^, mmHg**	73 (65–84)	72 (66–91)	0.621
**PEEP ^b^, mbar**	15 (11–16)	13 (11–15)	0.284
**Pinsp ^b^, mbar**	30 (27–35)	33 (27–36)	0.215
**Tidal volume ^b^, mL**	381 (303–454)	347 (287–449)	0.33
**Respiratory rate ^b^, breaths/min**	28 (25–31)	30 (26–32)	0.154
**Days with ECMO**	12 (6–21)	7 (5–14)	0.028
**Days from ECMO start to discharge**	24 (14–40)	10 (5–26)	<0.001
**Length of ICU stay, days**	27 (15–45)	17 (10–30)	0.005
**ICU mortality**	92 (57.1)	36 (83.7)	0.001

Demographic and clinical characteristics of the study population stratified by the diagnosis of intracranial hemorrhage. ECMO: extracorporeal membrane oxygenation. COPD: chronic obstructive pulmonary disease. AIDS: acquired immune deficiency syndrome. SAPS II: Simplified Acute Physiology Score. SOFA: Sequential organ Failure Assessment. RRT: renal replacement therapy. ICU: intensive care unit. ASS: acetylsalicylic acid. ^a^ Vital signs were measured before ECMO start. ^b^ Ventilation settings before ECMO start. Data are presented as *n* (%) or median with 25th and 75th percentiles. The bold is differentiate between main variables and subcategories (main category: “liver disease”; subcategory: “mild”, “moderate to severe”).

**Table 2:** Types of intracranial hemorrhage.

	Intracranial Hemorrhage	No COVID-19	COVID-19	*p* ^a^
	(*n* = 43)	(*n* = 26)	(*n* = 17)	
**ICH**	29 (67.4)	12 (46.2)	17 (100.0)	0.464
**Berlin classification ^b^**				0.993
*I/A*	19 (44.2)	8 (30.8)	11 (64.7)	
*II/B*	3 (7.0)	0 (0.0)	3 (17.6)	
*III/C*	7 (16.3)	4 (15.4)	3 (17.6)	
**IVH**	18 (41.9)	10 (38.5)	8 (47.1)	0.738
**EDH/SDH/SAH**	16 (37.2)	8 (30.8)	8 (47.1)	0.666
***Volumes of ICH and IVH***				
**Volume of ICH, mL**	55 (21–105)	69 (53–120)	45 (20–81)	0.250
**Volume of IVH, mL**	44 (30–51)	39 (30–51)	45 (21–92)	1.000

Characteristics of intracranial hemorrhage in all patients (*n* = 43) and stratified by the presence of COVID-19. ^a^ *p*-values refer to the statistical difference in characteristics between patients with and without COVID-19. ^b^ Classification of intraparenchymal hemorrhages (ICH) during extracorporeal membrane oxygenation according to Prinz et al. (6). Data are presented as *n* (%) or median with 25th and 75th percentiles. IVH: intraventricular hemorrhage. EDH: epidural hematoma. SDH: subdural hematoma. SAH: subarachnoid hemorrhage. Bold words characterize main categories.

**Table 3:** Cox regression for association with intracranial hemorrhage.

	Initial (Full) Model			Final (Stepwise-Backward) Model			
	HR	95% CI	*p*	HR	95% CI		*p*
COVID-19	3.503	1.178; 10.412	0.024	2.945	1.079; 8.038		0.035
Year of ECMO implantation	0.995	0.818; 1.209	0.957	1.012	0.839; 1.221		0.902
Sex	0.859	0.431; 1.713	0.667				
Charlson Comorbidity Index (ln)	0.854	0.489; 1.492	0.579				
Carboxyhemoglbin (ln) ^a^, %	0.372	0.142; 0.974	0.044	0.330	0.135; 0.806		0.015
ΔPaCO_2_ (ln) ^b^, mmHg	1.177	0.647; 2.140	0.596				
Platelets (ln) ^c^, 10^9^/L	0.996	0.592; 1.675	0.988				
Fibrinogen ^c^, g/L	1.320	0.984; 1.769	0.064				
aPTT (ln) ^d^, s	1.364	0.527; 3.531	0.523				
Cannula size (max), Fr	0.952	0.785; 1.155	0.618				
SOFA on admission	0.938	0.857; 1.027	0.166				

Cox regression with independent variables that were selected based on clinical considerations, time between ECMO start and diagnosis of intracranial hemorrhage as response variable, and intracranial hemorrhage as the dependent variable. ^a^ Highest value during extracorporeal membrane oxygenation (ECMO) or before intracranial hemorrhage. ^b^ Difference between the last arterial partial pressure of carbon dioxide (PaCO_2_) before ECMO start and PaCO_2_ 24 h after ECMO start. ^c^ Lowest value during ECMO or before intracranial hemorrhage. ^d^ Activated partial thromboplastin time, highest value during ECMO or before intracranial hemorrhage. The variables Charlson Comorbidity Index, ΔPaCO_2_, carboxyhemoglobin, activated partial thromboplastin time, and platelets were transformed to their natural logarithm, because they were right-skewed. SOFA: Sequential Organ Failure Assessment.

## Data Availability

The data presented in this study are available on request from the corresponding author. The data are not publicly available due to the General Data Protection Regulation.

## Supplementary Materials

The following are available online at https://www.mdpi.com/article/10.3390/jcm11010028/s1, Figure S1: Probability of survival by the type of intraparenchymal hemorrhage. Figure S2: Probability of intracranial hemorrhage during extracorporeal membrane oxygenation; Figure S3: Examples of intraparenchymal hemorrhage; Table S1: Blood gas analyses before and after implantation of extracorporeal membrane oxygenation; Table S2: Vital signs, laboratory and arterial blood gas parameters, settings of ventilation and extracorporeal membrane oxygenation at time of diagnosis of intracranial hemorrhage; Table S3: Sensitivity analysis; Table S4: Characteristics of patients with intracranial hemorrhage; Additional Text S1: Management of venovenous extracorporeal membrane oxygenation (vv-ECMO) for acute respiratory distress syndrome and data collection.
