# Supplementary material for: Association of COVID-19 with Intracranial Hemorrhage during Extracorporeal Membrane Oxygenation for Acute Respiratory Distress Syndrome: A 10-Year Retrospective Observational Study
Source: J Clin Med. 2021 Dec 22;11(1):28. doi: 10.3390/jcm11010028 (PMC8745037; doi:10.3390/jcm11010028)
Supplement: Supplementary file 1 [file jcm-11-00028-s001.zip › Additional Text S1.pdf]

Additional Text S1

Management of venovenous extracorporeal membrane oxygenation (vv-ECMO) for acute respiratory distress syndrome

For cannulation jugular and femoral veins were accessed. All cannulas were placed percutaneously after vessel puncture with Seldinger technique by a cardiac surgeon or an experienced intensivist. Cannula sizes were chosen according to the vessel diameter as identified by ultrasound (13-25 Fr). Blood flow was titrated to maintain arterial partial pressure of oxygen between 55 and 65 mmHg or arterial oxygen saturation ≥90%. The sweep gas flow was chosen to ensure removal of carbon dioxide with a pH between 7.25 and 7.4. Upon start of vv-ECMO a reduction in arterial partial pressure of carbon dioxide not higher than 20 mmHg was aimed at.

During cannulation a bolus of unfractionated heparin (5000 IE) was administered. The effect of heparin was measured with the activated clotting time during cannulation and start of vv-ECMO. Thereafter, unfractionated heparin was administered continuously and monitored with the partial thromboplastin time (pTT). Target pTT was 40 to 50 seconds in all patients. The HIT 4T score was used to screen for heparin-induced thrombocytopenia.[1] In patients with a HIT score ≥4 an immunoassay was performed. In case of laboratory-confirmed heparin-induced thrombocytopenia argatroban was administered continuously for anticoagulation.

Platelets were transfused in the presence of severe thrombocytopenia (<70,000 G/L). Fibrinogen levels were maintained above 1.5 g/L.

For circulatory support, patients were treated with restrictive fluid management after initial resuscitation and continuous norepinephrine to maintain a mean arterial pressure of ≥65 mmHg. If required, sedation was titrated to achieve a score of 0 or -1 on the Richmond Agitation and Sedation Scale (RASS). In patients who required neuromuscular blockade to

---

[1] G K Lo, D Juhl, T E Warkentin, C S Sigouin, P Eichler, A Greinacher: Evaluation of pretest clinical score (4 T's) for the diagnosis of heparin-induced thrombocytopenia in two clinical settings. J Thromb Haemost. 2006 Apr;4(4):759-65.

facilitate mechanical ventilation and to reduce patient-ventilator dyssynchrony or in patients with severe hemodynamic instability, lower RASS scores were aimed at. For sedation, propofol, midazolam, isoflurane, dexmedetomidine or a combination of these substances were used.

Data collection

Data collection for demographic and clinical baseline characteristics, the Simplified Acute Physiology Score (SAPS) II, clinical management of ARDS, laboratory parameters, blood gas analyses, settings of ventilation and ECMO, and vital signs was completed with reference to the individual electronic semi-automatic health record system (Integrated Care Manager, Version 10.01, Dräger AG, Lübeck, Germany). Data were anonymized upon retrieval. The participants' Charlson Comorbidity Index scores were determined based on their medical histories. We calculated their Sepsis-related Organ Failure Assessment (SOFA) scores on admission. Additionally, the duration of mechanical ventilation and the need for continuous renal replacement therapy during their ICU stays were documented. We reviewed every case for ICU mortality; for survivors, the place of discharge was documented.
