# Supplementary material for: Association of COVID-19 with Intracranial Hemorrhage during Extracorporeal Membrane Oxygenation for Acute Respiratory Distress Syndrome: A 10-Year Retrospective Observational Study
Source: J Clin Med. 2021 Dec 22;11(1):28. doi: 10.3390/jcm11010028 (PMC8745037; doi:10.3390/jcm11010028)
Supplement: Supplementary file 1 [file jcm-11-00028-s001.zip › R1_Table_S1_131221.pdf]

Table S1 – Blood gas analyses before and after implantation of extracorporeal membrane oxygenation

Arterial blood gas parameter	No intracranial hemorrhage (n=161)	Intracranial hemorrhage (n=43)	*p*
**PaO_2 before ECMO, mmHg**	75 (61-96)	70 (64-85)	0.539
**$PaCO_2$ before ECMO, mmHg**	62 (49-81)	66 (57-84)	0.200
**pH before ECMO**	7.26 (7.16-7.36)	7.27 (7.17-7.39)	0.943
**HCO_3^- before ECMO, mmol/L**	26 (21-30)	26 (23-30)	0.333
**Lactate before ECMO, mmol/L**	1.8 (1.1-3.3)	2.7 (1.3-4.5)	0.245
**Hemoglobin before ECMO, mg/dL**	10.0 (8.3-12.0)	9.5 (8.5-11.6)	0.340
**Glucose before ECMO, mg/dL**	141 (109-182)	146 (121-170)	0.960
**1st PaO_2 after ECMO start, mmHg**	96 (73-172)	80 (68-104)	0.059
**1st $PaCO_2$ after ECMO start, mmHg**	45 (38-55)	52 (45-61)	0.002
**1st pH after ECMO start**	7.37 (7.28-7.46)	7.34 (7.25-7.43)	0.302
**1st HCO_3^- after ECMO start, mmol/L**	25 (21-31)	28 (24-33)	0.155
**1st lactate after ECMO start, mmol/L**	2.2 (1.4-4.1)	2.9 (1.7-5.2)	0.214
**1st hemoglobin after ECMO start, mg/dL**	9.5 (8.2-11.1)	8.9 (7.9-10.3)	0.091
**1st glucose after ECMO start, mg/dL**	142 (107-178)	137 (116-179)	0.682
**2nd PaO_2 after ECMO start, mmHg**	75 (65-97)	72 (57-91)	0.112
**2nd $PaCO_2$ after ECMO start, mmHg**	43 (37-53)	50 (44-63)	<0.001
**2nd pH after ECMO start**	7.39 (7.32-7.46)	7.37 (7.30-7.42)	0.237
**2nd HCO_3^- after ECMO start, mmol/L**	25 (20-31)	28 (25-32)	0.114
**2nd lactate after ECMO start, mmol/L**	2.5 (1.7-4.8)	3.4 (1.8-4.9)	0.381
**2nd hemoglobin after ECMO start, mg/dL**	10.0 (8.7-11.1)	9.2 (8.3-10.5)	0.079
**2nd glucose after ECMO start, mg/dL**	143 (104-179)	141 (112-180)	0.728
**3rd PaO_2 after ECMO start, mmHg**	75 (64-91)	72 (65-92)	0.906
**3rd $PaCO_2$ after ECMO start, mmHg**	42 (38-49)	49 (45-58)	<0.001
**3rd pH after ECMO start**	7.38 (7.30-7.46)	7.37 (7.28-7.42)	0.304
**3rd HCO_3^- after ECMO start, mmol/L**	25 (21-31)	28 (22-31)	0.165
**3rd lactate after ECMO start, mmol/L**	2.6 (1.6-5.1)	3.3 (1.9-6.2)	0.311
**3rd hemoglobin after ECMO start, mg/dL**	10.3 (9.2-11.2)	9.8 (8.8-10.8)	0.104
**3rd glucose after ECMO start, mg/dL**	148 (109-193)	146 (114-178)	0.719
**PaO_2 24 h after ECMO start, mmHg**	71 (65-81)	71 (61-85)	0.772
**$PaCO_2$ 24 h after ECMO start, mmHg**	45 (40-49)	49 (44-54)	0.005
**pH 24 h after ECMO start**	7.41 (7.36-7.45)	7.41 (7.36-7.45)	0.880
**HCO_3^- 24 h after ECMO start, mmol/L**	28 (23-32)	29 (26-31)	0.192
**Lactate 24 h after ECMO start, mmol/L**	1.9 (1.2-3.4)	1.9 (1.3-2.8)	0.919
**Hemoglobin 24 h after ECMO start, mg/dL**	10.3 (9.6-11.2)	10.3 (9.3-11.0)	0.617
**Glucose 24 h after ECMO start, mg/dL**	147 (122-178)	153 (118-178)	0.917
**1st $\Delta PaCO_2$[a], mmHg**	14 (5-28)	13 (6-28)	0.720
**$\Delta PaCO_2$ 24 h[b], mmHg**	17 (6-33)	22 (6-36)	0.855
**Mean $PaCO_2$ during ECMO, mmHg**	45 (42-48)	48 (44-52)	0.001

**$PaCO_2$ variability[c] during ECMO, mmHg**	33 (24-47)	39 (29-69)	0.073
**Mean pH during ECMO**	7.41 (7.38-7.44)	7.42 (7.38-7.45)	0.656
**pH variability[c] during ECMO**	0.00 (0.00-0.01)	0.00 (0.00-0.01)	0.441

Table S1: Results from arterial blood gas analyses stratified by the diagnosis of intracranial hemorrhage during venovenous extracorporeal membrane oxygenation (ECMO). “Before ECMO” refers to the final blood gas analysis before ECMO implantation. 1st, 2nd, and 3rd blood gas analyses refer to the first three analyses after ECMO start. PaO_2: arterial partial pressure of oxygen. $PaCO_2$: arterial partial pressure of carbon dioxide. HCO_3^-: bicarbonate. [a]Difference between the last $PaCO_2$ before ECMO start and the first $PaCO_2$ after ECMO initiation. [b]Difference between the last $PaCO_2$ before ECMO start and $PaCO_2$ 24 h after ECMO initiation [c]The variability of $PaCO_2$ and pH was calculated as the variance of all $PaCO_2$/pH values during venovenous ECMO support. Data are presented as n (%) or median with 25th and 75th percentiles.
