# Supplementary material for: Association of COVID-19 with Intracranial Hemorrhage during Extracorporeal Membrane Oxygenation for Acute Respiratory Distress Syndrome: A 10-Year Retrospective Observational Study
Source: J Clin Med. 2021 Dec 22;11(1):28. doi: 10.3390/jcm11010028 (PMC8745037; doi:10.3390/jcm11010028)
Supplement: Supplementary file 1 [file jcm-11-00028-s001.zip › R1_Table_S3.pdf]

Table S3 – Sensitivity analysis

Independent variable	Years 2020 - 2021					
	Initial (full) model			Final (stepwise-backward) model		
	HR	95% CI	*p*	HR	95% CI	*p*
**COVID-19**	0.187	0.031;1.120	0.066			
**Gender**	0.526	0.138;2.005	0.347			
**Charlson Comorbidity Index (ln)**	0.270	0.044;1.654	0.157			
**Carboxyhemoglobin (ln)[a], %**	0.009	0.001;0.115	<0.001	0.016	0.002;0.123	<0.001
**Δ$PaCO_2$ (ln)[b], mmHg**	3.918	0.432;35.533	0.225			
**Platelets (ln)[c], 10^9/L**	0.929	0.218;3.963	0.921			
**Fibrinogen[c], g/L**	14.885	2.619;84.599	0.002	7.708	2.238;26.549	0.001
**aPTT (ln)[d], sec**	2.879	0.347;23.875	0.327			
**Cannula size (max), Fr**	1.685	0.8993.161	0.720			
**SOFA on admission**	0.767	0.628;0.937	0.009	0.840	0.735;0.959	0.010
**Year of ECMO implantation**	1.862	0.290;11.962	0.513			

Table S3: Cox regression with independent variables that were selected based on clinical considerations and intracranial hemorrhage as the dependent variable. The model was rerun for years 2020-2021. [a]Highest value during extracorporeal membrane oxygenation (ECMO) or before intracranial hemorrhage. [b]Difference between the last arterial partial pressure of carbon dioxide ($PaCO_2$) before ECMO start and $PaCO_2$ 24 h after ECMO start. [c]Lowest value during ECMO or before intracranial hemorrhage. [d]Highest value during ECMO or before intracranial hemorrhage. The variables Charlson Comorbidity Index, Δ$PaCO_2$, carboxyhemoglobin, activated partial thromboplastin time (aPTT), and platelets were transformed to their natural logarithm, because they were right-skewed. SOFA: Sequential Organ Failure Assessment.
