# Supplementary material for: Association of COVID-19 with Intracranial Hemorrhage during Extracorporeal Membrane Oxygenation for Acute Respiratory Distress Syndrome: A 10-Year Retrospective Observational Study
Source: J Clin Med. 2021 Dec 22;11(1):28. doi: 10.3390/jcm11010028 (PMC8745037; doi:10.3390/jcm11010028)
Supplement: Supplementary file 1 [file jcm-11-00028-s001.zip › R1_Table_S4_131221.pdf]

Table S4 – Characteristics of patients with intracranial hemorrhage

	No COVID-19 (n=26)	COVID-19 (n=17)
Demographic and clinical characteristics		
**Age, years**	55 (46-61)	58 (51-65)
**Gender, (female)**	9 (34.6)	6 (35.3)
**Body Mass index**	25.1 (21.4-30.8)	31.9 (26.1-35.1)
**Charlson Comorbidity Index**	3 (1-4)	2 (1-2)
**SOFA on admission**	12 (9-14)	10 (8-12)
**SAPS II on admission**	40 (36-52)	42 (36-50)
**Anticoagulation or coagulopathy before ECMO**		
no	20 (76.9)	13 (76.5)
yes	6 (23.1)	3 (17.6)
unknown	0 (0.0)	1 (5.9)
**ASS before ECMO**	5 (19.2)	2 (11.8)
**Platelet inhibitor before ECMO**		
no	25 (96.2)	17 (100)
clopidogrel	0 (0.0)	0 (0.0)
prasugrel	1 (3.8)	0 (0.0)
ticagrelor	0 (0.0)	0 (0.0)
**Duration of mechanical ventilation, days**	13 (8-24)	16 (9-30)
**Nitric oxide**	15 (57.7)	10 (58.8)
**RRT**	20 (76.9)	9 (52.9)
**RRT duration, days**	11 (4-20)	1 (0-16)
**Device**		
venovenous ECMO	21 (80.8)	15 (88.2)
iLA activve® (pump assisted)	5 (19.2)	2 (11.8)
**Minimum cannula size**	17 (17-19)	17 (17-19)
**Maximum cannula size**	24 (23-25)	24 (24-25)
**Days from ICU admission to ICRH**	10 (6-18)	9 (1-17)
**Days from ECMO start to ICRH**	7 (3-13)	6 (3-11)
**ICU mortality**	21 (80.8)	15 (88.2)
Blood gas analyses before and after ECMO start		
**PaO_2 before ECMO, mmHg**	75 (66-87)	70 (64-79)
**$PaCO_2$ before ECMO, mmHg**	67 (57-86)	63 (57-82)
**pH before ECMO**	7.27 (7.15-7.35)	7.27 (7.19-7.39)
**HCO_3^- before ECMO, mmol/L**	26 (24-28)	29 (23-32)
**Lactate before ECMO, mmol/L**	3 (1-5)	3 (2-3)
**Hemoglobin before ECMO, mg/dL**	10 (9-12)	9 (8-12)

**Glucose before ECMO, mg/dL**	147 (120-156)	145 (126-185)
**1st PaO_2 after ECMO start, mmHg**	83 (63-104)	78 (70-91)
**1st $PaCO_2$ after ECMO start, mmHg**	54 (47-61)	52 (45-61)
**1st pH after ECMO start**	7.33 (7.25-7.42)	7.37 (7.26-7.45)
**1st HCO_3^- after ECMO start, mmol/L**	27 (24-30)	29 (24-33)
**1st lactate after ECMO start, mmol/L**	3 (1-5)	4 (2-5)
**1st hemoglobin after ECMO start, mg/dL**	9 (8-10)	9 (8-11)
**1st glucose after ECMO start, mg/Dl**	128 (105-157)	152 (136-199)
**2nd PaO_2 after ECMO start, mmHg**	65 (55-87)	78 (71-92)
**2nd $PaCO_2$ after ECMO start, mmHg**	49 (44-62)	53 (46-63)
**2nd pH after ECMO start**	7.34 (7.31-7.44)	7.37 (7.30-7.42)
**2nd HCO_3^- after ECMO start, mmol/L**	26.5 (25.1-30.4)	28.9 (24.5-31.9)
**2nd lactate after ECMO start, mmol/L**	3.1 (1.5-4.9)	3.7 (1.9-4.8)
**2nd hemoglobin after ECMO start, mg/dL**	9.4 (8.5-10)	9.1 (8.2-11.1)
**2nd glucose after ECMO start, mg/dL**	133 (91-180)	143 (124-200)
**3rd PaO_2 after ECMO start, mmHg**	70 (64-79)	79 (71-97)
**3rd $PaCO_2$ after ECMO start, mmHg**	48 (44-53)	51 (45-58)
**3rd pH after ECMO start**	7.36 (7.28-7.41)	7.37 (7.28-7.42)
**3rd HCO_3^- after ECMO start, mmol/L**	26.4 (22.2-29.9)	28.4 (24.4-30.5)
**3rd lactate after ECMO start, mmol/L**	3.1 (2-6.2)	3.7 (1.8-5.6)
**3rd hemoglobin after ECMO start, mg/dL**	9.8 (8.8-10.6)	9.3 (8.9-11.4)
**3rd glucose after ECMO start, mg/dL**	145 (114-171)	146 (115-202)
**PaO_2 24 h after ECMO start, mmHg**	73 (61-86)	68 (66-83)
**$PaCO_2$ 24 h after ECMO start, mmHg**	49 (42-52)	49 (45-57)
**pH 24 h after ECMO start**	7.41 (7.36-7.46)	7.40 (7.36-7.44)
**HCO_3^- 24 h after ECMO start, mmol/L**	28.4 (25.4-30.1)	29.6 (27.9-31.4)
**Lactate 24 h after ECMO start, mmol/L**	2.0 (1.2-3.6)	1.9 (1.3-2.2)
**Hemoglobin 24 h after ECMO start, mg/dL**	10.2 (9.7-10.9)	10.5 (8.9-11.0)
**Glucose 24 h after ECMO start, mg/dL**	154 (119-173)	152 (118-184)
Parameters at time of intracranial hemorrhage		
**SAPS II**	49 (38-54)	46 (39-47)
**Pump speed, rpm**	3535 (3155-4614)	4151 (3465-4698)
**Blood flow, L/min**	4.10 (3.60-4.75)	4.80 (4.15-5.44)
**Sweep gas flow, L/min**	4.0 (3.0-6.0)	4.8 (3.5-6.0)
**SBP 1, mmHg**	124.5 (114-135)	124.5 (117-141)
**DBP 1, mmHg**	56.5 (49-66)	60.5 (55.5-63.5)
**MAP 1, mmHg**	82.0 (69.0-90.0)	81.5 (74.5-87.5)
**SBP 2, mmHg**	120.0 (102-135.0)	119.0 (111.5-133.0)
**DBP 2, mmHg**	55.5 (47.0-61.0)	56.0 (52.0-61.5)

**MAP 2, mmHg**	75.5 (62.0-83.0)	74.5 (71.5-85)
**SBP 3, mmHg**	124.5 (104.0-138.0)	111.5 (107.0-131.5)
**DBP 3, mmHg**	55.5 (47.0-62.0)	55.5 (50.0-61.0)
**MAP 3, mmHg**	76.5 (64.0-82.0)	75.0 (67.0-81.0)
**aPTT, sec**	49.6 (43.1-59.5)	54 (47-61)
**Fibrinogen, g/L**	4.70 (3.66-4.97)	4.72 (3.75-5.44)
**Platelets, 10^9/L**	102.5 (61-161)	124 (88-136)
**Quick, %**	84 (64-98)	94 (70-99)
**Antithrombin III activity, %**	80 (56-97)	86 (65-103)
**Highest carboxyhemoglobin until ICRH**	2.95 (2.10-3.50)	3.20 (2.70-3.90)
**Lowest ATIII until ICRH**	63.8 (48.0-73.5)	70.0 (63.0-86.0)
**Highest aPTT until ICRH**	102.8 (81.0-160.1)	105.0 (72.0-151.0)
**Lowest fibrinogen until ICRH**	3.23 (2.0-4.22)	4.01 (3.64-4.87)
**Lowest quick until ICRH**	52 (36-69)	65 (54-90)
**Lowest platelets until ICRH**	70 (54-104)	86 (61-115)
**PaO_2 before ICRH 1, mmHg**	70.9 (61.5-81.4)	72.9 (67.6-87.8)
**$PaCO_2$ before ICRH 1, mmHg**	47.7 (41.5-54.8)	46.9 (43.8-52.1)
**pH before ICRH 1**	7.40 (7.35-7.50)	7.44 (7.38-7.47)
**HCO_3^- before ICRH 1, mmol/L**	28.4 (27.0-30.6)	31 (24.9-33.8)
**Lactate before ICRH 1, mmol/L**	1.4 (1.0-2.1)	1.3 (1.0-1.91)
**Hemoglobin before ICRH 1, g/dL**	9.7 (9.3-10.5)	10.1 (8.9-10.4)
**Glucose before ICRH 1, mg/dL**	146.0 (123.0-159.0)	145.5 (134.0-159.5)
**PaO_2 before ICRH 2, mmHg**	72.9 (59.4-90.6)	76.4 (65.5-86.1)
**$PaCO_2$ before ICRH 2, mmHg**	46.2 (42.7-53.2)	47 (44.1-51.8)
**PH before ICRH 2**	7.41 (7.36-7.48)	7.43 (7.36-7.48)
**HCO_3^- before ICRH 2, mmol/L**	28.7 (27.0-30.4)	30.0 (24.9-33.0)
**Lactate before ICRH 2, mmol/L**	1.4 (1.0-2.3)	1.3 (0.9-1.7)
**Hemoglobin before ICRH 2, g/dL**	9.8 (9.2-10.9)	9.7 (8.8-10.3)
**Glucose before ICRH 2, mg/dL**	144.0 (123.0-160.0)	154.5 (133.0-174.5)
**PaO_2 before ICRH 3, mmHg**	77.4 (60.5-88.7)	72.1 (66.8-78.6)
**$PaCO_2$ before ICRH 3, mmHg**	46.4 (42.1-54.5)	48.1 (43.5-51.4)
**pH before ICRH 3**	7.42 (7.35-7.50)	7.42 (7.36-7.48)
**HCO_3^- before ICRH 3, mmol/L**	28.8 (26.8-30.6)	30.0 (25.6-34.4)
**Lactate before ICRH 3, mmol/L**	1.3 (1.1-2.3)	1.3 (0.9-1.7)
**Hemoglobin before ICRH 3, g/dL**	9.9 (8.8-10.8)	9.9 (8.9-10.4)
**Glucose before ICRH 3, mg/dL**	144.0 (129.0-160.0)	130.5 (116.0-161.0)

Table S4: Demographic and clinical characteristics, blood gas analyses before and after ECMO start of patients with intracranial hemorrhage stratified by the presence of COVID-19. Vital signs, blood gas analyses, settings of extracorporeal membrane oxygenation (ECMO), and coagulation parameters at the time of diagnosis of intracranial hemorrhage (ICRH). Data are presented as n (%) or median with 25th and 75th percentiles. “Before ECMO” refers to the final blood gas analysis before extracorporeal membrane oxygenation (ECMO) implantation. 1st, 2nd, and 3rd blood gas analyses refer to the first three analyses after ECMO start. Blood pressure values refer to the last three measurements before the diagnosis of ICRH. Blood gas analyses refer to the last three analyses before the diagnosis of ICRH. ECMO: extracorporeal membrane oxygenation. SAPS II: Simplified Acute Physiology Score. SOFA: Sequential organ Failure Assessment. ASS: acetylsalicylic acid. RRT: renal replacement therapy. ICU: intensive care unit. PaO_2: arterial partial pressure of oxygen. $PaCO_2$: arterial partial pressure of carbon dioxide. HCO_3^-: bicarbonate. SBP: systolic blood pressure. DBP: diastolic blood pressure. MAP: mean arterial pressure. aPTT: Activated Partial Thromboplastin Time.
