# Supplementary material for: Association of COVID-19 with Intracranial Hemorrhage during Extracorporeal Membrane Oxygenation for Acute Respiratory Distress Syndrome: A 10-Year Retrospective Observational Study
Source: J Clin Med. 2021 Dec 22;11(1):28. doi: 10.3390/jcm11010028 (PMC8745037; doi:10.3390/jcm11010028)
Supplement: Supplementary file 1 [file jcm-11-00028-s001.zip › Supplementary_figures_legends_151221.pdf]

Figure S1

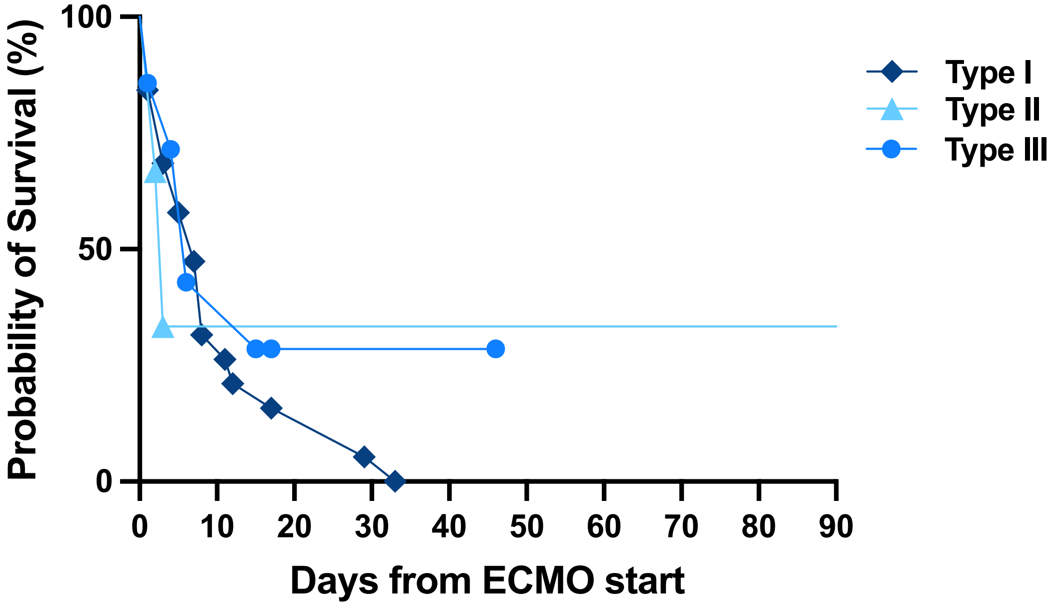

Number at risk

**Day**	0	11	12	15	17	29	33	46	161
**Type I**	19	6	5	---	4	3	1	---	---
**Type II**	3	---	---	---	---	---	---	---	1
**Type III**	7	---	---	3	2	---	---	1	---

Figure S1: Probability of survival from initiation of extracorporeal membrane oxygenation by type of intracerebral hemorrage. Type I: lobar hemorrhage ± intraventricular hemorrhage. Type II: localized lobar hemorrhage without intraventricular hemorrhage. Type III: multiple small lesions.

Figure S2

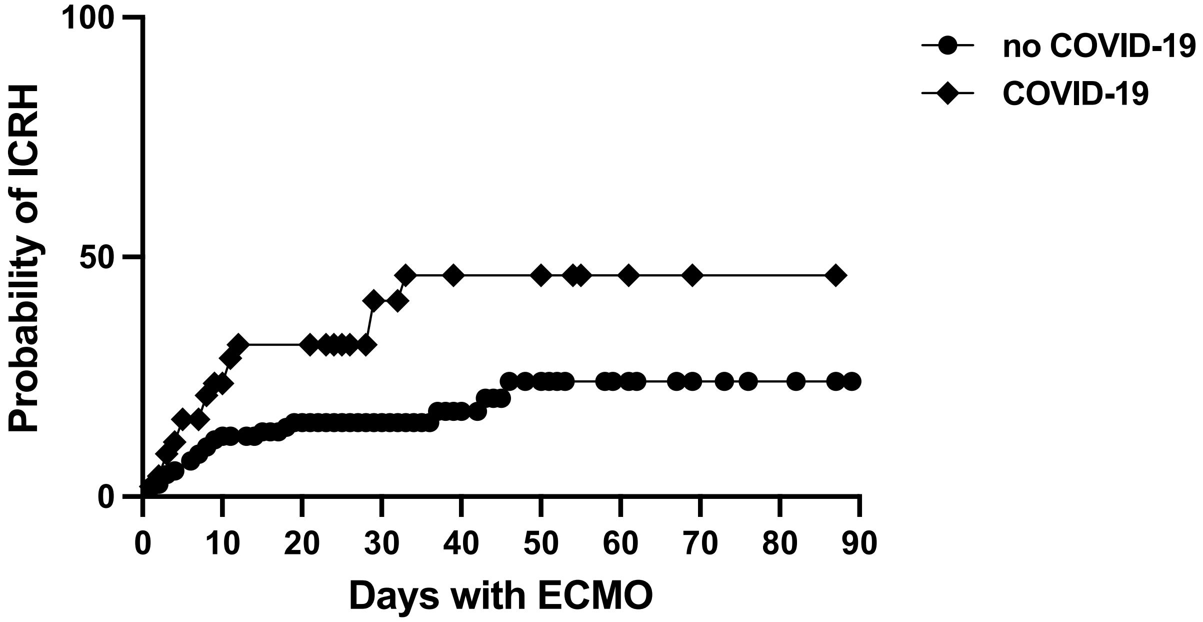

Number at risk

**Day**	0	30	61	89
**no COVID-19**	154	47	12	4
**COVID-19**	47	---	4	---

Figure S2: Probability of intracranial hemorrhage (ICRH) from initiation of extracorporeal membrane oxygenation (ECMO) by the presence of COVID-19.

Figure S3

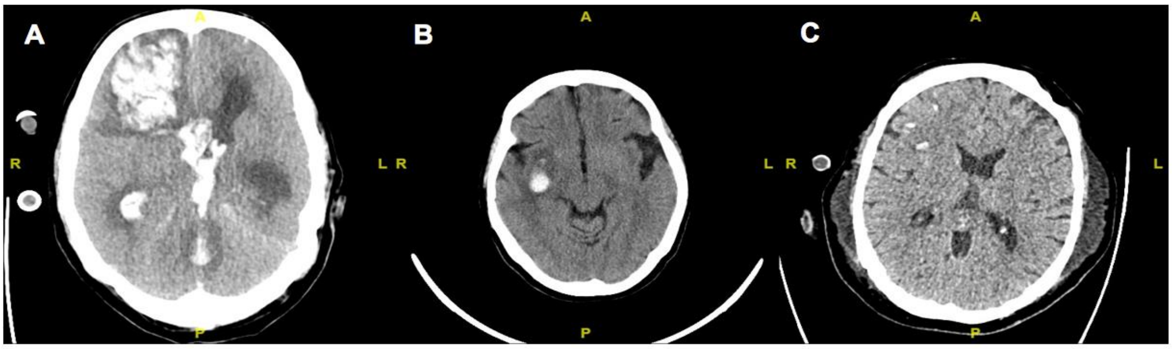

Figure S3: Examples of type I (A: lobar bleeding with intraventricular hemorrhage), type II (B: localized lobar bleeding without intraventricular hemorrhage), and type III (C: multiple small bleedings) hemorrhage.
