# Supplementary material for: Association of COVID-19 with Intracranial Hemorrhage during Extracorporeal Membrane Oxygenation for Acute Respiratory Distress Syndrome: A 10-Year Retrospective Observational Study
Source: J Clin Med. 2021 Dec 22;11(1):28. doi: 10.3390/jcm11010028 (PMC8745037; doi:10.3390/jcm11010028)
Supplement: Supplementary file 1 [file jcm-11-00028-s001.zip › Table S2.pdf]

Table S2 – Vital signs, laboratory and arterial blood gas parameters, settings ventilation and extracorporeal membrane oxygenation at time of diagnosis of intracranial hemorrhage

Variable	Intracranial hemorrhage (n=43)
**SAPS II**	46 (38-52)
**Pump speed, rpm**	3700 (3265-4620)
**Blood flow, L/min**	4.35 (3.8-5.04)
**Sweep gas flow, L/min**	4.0 (3.5-6.0)
**FiO_2**	1.0 (1.0-1.0)
**Inspiratory pressure, mbar**	24 (18-27)
**Positive end-expiratory pressure, mbar**	11 (9-13)
**Tidal volume, mL**	247 (144-369)
**Respiratory rate, breaths/min**	29 (15-27)
**SBP 1, mmHg**	125 (115-138)
**DBP 1, mmHg**	58 (52-66)
**MAP 1, mmHg**	82 (74-88)
**SBP 2, mmHg**	119 (109-135)
**DBP 2, mmHg**	56 (48-61)
**MAP 2, mmHg**	75 (71-84)
**SBP 3, mmHg**	121 (106-137)
**DBP 3, mmHg**	56 (49-62)
**MAP 3, mmHg**	75 (67-82)
**Noradrenaline, µg/kg/min[a]**	0.25 (0.11-0.46)
**Activated Partial Thromboplastin Time, sec**	53 (46-60)
**Fibrinogen, g/L**	4.7 (3.7-5.4)
**Platelets, 10^9/L**	107 (68-156)
**Quick, %**	85 (64-99)
**Antithrombin III activity, %**	84 (60-102)
**PaO_2 1, mmHg**	72 (62-87)
**$PaCO_2$ 1, mmHg**	48 (43-54)
**pH 1**	7.41 (7.35-7.48)
**HCO_3^- 1, mmol/L**	28 (26-33)
**Lactate 1, mmol/L**	1.3 (1.0-2.1)
**Hemoglobin 1, g/dL**	9.7 (9.0-10.4)
**Glucose 1, mg/dL**	9.7 (9.0-10.4)
**PaO_2 2, mmHg**	74 (61-88)
**$PaCO_2$ 2, mmHg**	46 (44-53)
**pH 2**	7.42 (7.36-7.48)
**HCO_3^- 2, mmol/L**	29 (27-33)
**Lactate 2, mmol/L**	1.4 (0.9-2.1)
**Hemoglobin 2, g/dL**	9.7 (8.8-10.7)
**Glucose 2, mg/dL**	148 (125-164)

**PaO_2 3, mmHg**	73 (65-86)
**$PaCO_2$ 3, mmHg**	48 (42-52)
**pH 3**	7.42 (7.35-7.49)
**HCO_3^- 3, mmol/L**	29 (26-33)
**Lactate 3, mmol/L**	1.3 (1.0-2.2)
**Hemoglobin 3, g/dL**	9.9 (8.8-10.5)
**Glucose 3, mg/dL**	141 (120-160)

Table S2: Vital signs, settings of extracorporeal membrane oxygenation (ECMO) and mechanical ventilation, arterial blood gas analyses, and coagulation parameters at the time of diagnosis of intracranial hemorrhage. Blood pressure values refer to the last three measurements before ECMO start. Blood gas analyses refer to the last three analyses before ECMO start. [a]Highest dose of noradrenaline within 24 hours before diagnosis of intracranial hemorrhage. SAPS II: Simplified Acute Physiology Score. SBP: systolic blood pressure. DBP: diastolic blood pressure. MAP: mean arterial pressure. PaO_2: arterial partial pressure of oxygen. $PaCO_2$: arterial partial pressure of carbon dioxide. HCO_3^-: bicarbonate.
